# 89 Associations of active commuting and leisure-time physical activity with mental health symptoms among Finnish adults: a population-based study

**DOI:** 10.1093/eurpub/ckae114.149

**Published:** 2024-09-26

**Authors:** Kaija Appelqvist-Schmidlechner, Jenni Ervasti, Jaana I Halonen, Juuso J Jussila, Jouni Lahti, Timo Lanki, Santtu Mikkonen, Anna Pulakka, Paula Salo

**Affiliations:** Department of Public Health and Welfare, Finnish Institute for Health and Welfare (THL), Helsinki, Finland; Finnish Institute of Occupational Health; Department of Health Security, Finnish Institute for Health and Welfare (THL), Helsinki, Finland; Institute of Public Health and Clinical Nutrition, University of Eastern Finland, Kuopio, Finland; Department of Health Security, Finnish Institute for Health and Welfare (THL), Helsinki, Finland; Department of Public Health and Welfare, Finnish Institute for Health and Welfare (THL), Helsinki, Finland; Department of Public Health, University of Helsinki, Helsinki, Finland; Institute of Public Health and Clinical Nutrition, University of Eastern Finland, Kuopio, Finland; Department of Health Security, Finnish Institute for Health and Welfare (THL), Helsinki, Finland; Department of Environmental and Biological Sciences, University of Eastern Finland, Kuopio, Finland; Department of Environmental and Biological Sciences, University of Eastern Finland, Kuopio, Finland; Department of Public Health and Welfare, Finnish Institute for Health and Welfare (THL), Helsinki, Finland; Research Unit of Population Health, University of Oulu, Oulu, Finland; Department of Psychology and Speech-Language Pathology, University of Turku, Turku, Finland

## Abstract

**Purpose:**

Active commuting, that is, walking or cycling to work, can improve physical health. Yet, few studies have explored whether it could also enhance mental health. We examined cross-sectional associations of active commuting and, for comparison, leisure-time physical activity with depressive symptoms and psychological distress among Finnish adults.

**Methods:**

We included 3439 adults (mean age 45.0 years; 51% women) from the FinHealth 2017 Study. Based on their commuting and leisure-time physical activity behaviour, participants were categorised as active or passive commuters and as sedentary, recreationally active, or exercisers and athletes. Logistic regression was used to estimate odds ratios (OR), with 95% confidence intervals (CI), adjusting for major sociodemographic, health behaviour, and physical activity variables.

**Results:**

Active commuting, as a binary variable, was not associated with mental health outcomes compared to passive commuting. However, in dose-response analyses, high volumes of active commuting (≥ 30 minutes a day) were associated with higher odds of depressive symptoms (OR 1.58, 95% CI 1.18-2.13). Regarding leisure-time physical activity, recreationally active adults had lower odds of depressive symptoms (OR 0.57, 95% CI 0.44-0.73) and psychological distress (OR 0.46, 95% CI 0.32-0.68) compared to sedentary individuals. Similarly, exercisers and athletes had lower odds of depressive symptoms (OR 0.44, 95% CI 0.32-0.61) and lower odds of psychological distress (OR 0.34, 95% CI 0.21-0.55).

**Conclusions:**

We found leisure-time physical activity, but not active commuting, to be associated with lower odds of mental health symptoms. More research on commuting behaviour is needed to understand the complex relationship between active commuting and mental health.

